# ADAM10 is a key player in the diagnosis, prognosis and metastasis of non-small cell lung cancer (NSCLC)

**DOI:** 10.7150/jca.107236

**Published:** 2025-02-11

**Authors:** Wenqian Zhang, Liyao Yang, Mufan Li, Lianmei Zhang, Jingliang Cheng, Ali H. El-Far, Youhua Xu, Junjiang Fu

**Affiliations:** 1 Faculty of Chinese Medicine, Macau University of Science and Technology, Avenida Wai Long, Taipa, Macao 999078, China.; 2 Key Laboratory of Epigenetics and Oncology, the Research Center for Preclinical Medicine, Southwest Medical University, Luzhou 646000, Sichuan Province, China.; 3 Department of Oncology, The First People's Hospital of Loudi City, Loudi 417009, Hunan Province, China.; 4 Department of Pathology, The Affiliated Huaian No. 1 People's Hospital of Nanjing Medical University, Huai'an 223300, Jiangsu Province, China.

**Keywords:** DAM10, Non-small cell lung cancers, Prognostics

## Abstract

A disintegrin and metalloproteinase domain-containing protein 10 (ADAM10) plays critical roles in various cancer-associated biological events, such as cell multiplication, migration, and metastasis. This study employs both the TCGA database and patient samples to demonstrate that ADAM10 is highly expressed in non-small cell lung cancer (NSCLC) compared with normal tissue at different stages. Increased ADAM10 expression is positively correlated with decreased overall and recurrence-free survival. On the functional front, overexpression of ADAM10 promotes lung cancer cell progression, migration, and invasion, whereas downregulation of ADAM10 inhibits these processes. Mechanically, ADAM10 modulates the expression of Notch1, MMP9 and EMT markers such as Vimentin, N-cadherin, and E-cadherin. Overall, our findings suggest that ADAM10 may be a promising therapeutic and prognostic marker for NSCLC, emphasizing the importance of regulating its expression.

## 1. Introduction

Lung cancer, including non-small cell lung cancer (NSCLC), remains one of the most common and deadly forms of malignancy worldwide 1. Furthermore, despite significant improvements in surgical, chemical, radiation, and targeted therapies in recent years 2, the aggressive nature of NSCLC, characterized by high invasiveness and metastatic capability, frequently results in treatment failure and significantly impacts patient prognosis, contributing to low survival rates. As a result, identifying molecular targets capable of therapeutic intervention has become essential for treating these diseases and improving patient outcomes.

ADAM10, a transmembrane protein belonging to the ADAM family, plays crucial roles in regulating not only Notch receptor activity but also the cleavage of amyloid precursor protein, a key component initially discovered in Alzheimer's disease 3. This proteolytic property, facilitated by its metal-containing protease and cysteine-rich regions, enables ADAM10 to cut over 100 distinct substrates, including extracellular matrix proteins, cell adhesion molecules and growth factor receptors, such as amyloid precursor protein (APP), CD40L, Notch, EGFR/HER ligands, EGF, N-cadherin, L-selectin, programmed death-ligand 1 (PD-L1), Nectin-4, transmembrane activator and CAML interactor, and the stress-responsive proteins UL16-binding proteins 4-7. Consequently, ADAM10 is involved in diverse cellular processes, ranging from autoimmune responses and inflammation to apoptosis and cell metabolism 4-7. Its ability to cleave EphA5 has been linked to prostate cancer metastasis 8, and its contribution to uveal melanoma progression 9 further highlights its potential as an innovative target for cancer therapy 4, 10.

In the context of non-small cell lung cancer (NSCLC), such as the A549 cell line, reducing ADAM10 levels inhibits migration and invasion and prevents Notch1 protein cleavage 11. However, its sheddase activity as a biomarker for lung cancer must be further validated 12. Recently, by collecting protein mass spectrometry data from 74 stage I lung adenocarcinoma (LUAD) patients from Taiwan, Lu and colleagues 13 revealed five prognostic biomarkers, ADAM10, MAOA, MIF, TEK, and THBS2. Nevertheless, exploring new frontiers for ADAM10 in oncology is still necessary. To this end, the present study was designed. We observed increased ADAM10 levels in non-small cell lung cancer (NSCLC) tissue samples. Moreover, our investigation revealed that ADAM10 could indeed cleave specific substrate molecules, e.g., Notch1 and N-cadherin, thereby influencing the activation of Notch signaling and facilitating the progression of epithelial-to-mesenchymal transformation (EMT), ultimately driving tumor invasion and metastasis. These findings indicate that ADAM10 represents a promising therapeutic target for lung cancer, with potential applications in diagnosis, prognosis, and treatment.

## 2. Materials and methods

### 2.1 Differences in ADAM10 expression in non-small cell lung cancer

The TCGA pancancer data for lung cancer were retrieved from the UCSC database. (https://xenabrowser.net/). The dataset includes 60499 tumors and 10535 normal samples. Initially, the expression data of the ADAM10 (ENSG00000137845) gene in non-small cell lung cancer were extracted. After that, the tumor samples are separated into various datasets on the basis of subtype. Genes whose average expression was lower than one, including those with zero expression, were excluded.

We subsequently applied the R package to the data via the trimmed mean of the M values (TMM) method for normalization. The normalized data are then transformed to a log2 scale, with an additional value of one to avoid an undefined measure of log zero. R software (version 3.6.4) was used to calculate the differences in ADAM10 expression between tumor and normal samples and between non-small cell lung cancer (LUAD and LUSC) samples at different clinical stages. Unpaired Student's *t* test was used for the significance analysis of differences between pairs, and analysis of variance was adopted for the difference test among multiple groups of samples.

### 2.2 Survival analysis

The Kaplan‒Meier plotter (pancancer) database can be used to analyze the relationship between gene expression levels and survival in non-small cell lung cancer (NSCLC) patients and to conduct prognostic analysis for survival prediction 14. Experiments were carried out by selecting the Pan-Cancer column, entering “ADAM10”, choosing OS (overall survival)/RFS (recurrence-free survival), selecting “Lung Adenocarcinoma” and “Lung Squamous Carcinoma” for the cancer type, and making other default database selections, followed by conducting an analysis. The hazard ratio (HR), in conjunction with its 95% confidence intervals and the log-rank P value, was calculated.

### 2.3 Meta-analysis

A meta-analysis of lung cancer datasets was performed via the Lung Cancer Explorer (LCE, https://lce.biohpc.swmed.edu/lungcancer/) 15, which integrated gene profiling datasets of tumor patients and healthy subjects, which included more than 6,700 patients from the Gene Expression Omnibus (GEO), The Cancer Genomics Atlas (TCGA) and other published studies. The lung cancer tissue datasets containing LUAD (lung adenocarcinoma) and LUSC (lung squamous cell carcinoma) data were retrieved from the LCE dataset. For the meta-analysis of ADAM10 survival and expression levels in the retrieved datasets, the LUAD, LUSC, and normal groups had sample sizes exceeding 20. ADAM10 survival association meta-analysis in NSCLC was performed via the R package 'meta' to calculate the overall hazard ratio (HR) according to the HR of each dataset.

### 2.4 Cells and cell culture

Human lung cancer cell lines (H1299, H1975, A549, H460, and PC9) and normal human lung epithelial BEAS-2B cells were obtained from the American Type Culture Collection (ATCC), which uses Dulbecco's modified Eagle's medium (DMEM) supplemented with 10% FBS (Invigentech, cat#: A6803P, USA) and 1% penicillin‒streptomycin (Beyotime, China). The cells were cultured in 12- or 6-well plates. When the cell density reached approximately 50~60%, the ADAM10 inhibitor GI254023X (HY-19956, MedChem Express, USA) was added to each experimental group at concentrations of 0 and 20 μM for 24 h. In addition, when the cell density reached 40‒50%, each experimental group was transfected with plasmids (details of the transfection method are described in section 2.6) separately for subsequent experiments.

### 2.5 Lung cancer tissue collection

Paired lung cancer (LUAD or LUSC) tissues and adjacent nontumor histologically normal tissue samples were obtained from the Affiliated Huaian No. 1 People's Hospital of Nanjing Medical University. All patients had LUAD or LUSC with an age range of 45--74 years. Total proteins were extracted via 1 × EBC lysis buffer. Equal amounts of 2 × loading buffer were added, and the samples were mixed well, boiled and stored at -20 °C until use. This study was carried out in accordance with the Declaration of Helsinki (as revised in 2013) and received approval from the Ethics Committee of the Affiliated Huaian No. 1 People's Hospital of Nanjing Medical University.

### 2.6 Transfection assays

The pcDNA5-FRT-To-Vector plasmid was constructed, and the ADAM10 plasmid pCDNA5-FRT-To-ADAM10-2×FLAG was obtained from our laboratory 16, 17. Briefly, A549 and H1299 cells (approximately 0.1×10^6^ cells per well of a 12-well plate) were transfected with the pcDNA5-FRT-To-2×FLAG-Vect plasmid (pcDNA5vector) or pCDNA5-FRT-To-ADAM10-2×FLAG-Hyg plasmid (pcDNA5-ADAM10) via Lipofectamine 3000 (Life Technologies, USA) according to the manufacturer's protocol. Specifically, 0.1 µg of pcDNA5-Vector/ADAM10 was diluted in Opti-MEM^@^I(1X) Reduced-Serum Medium (Gibco, USA) per well to 0.3 µl of Lipofectamine 3000/P3000, and the mixture was incubated at room temperature for 5 min. Then, the two diluted solutions were gently mixed at room temperature for 20 min, and the mixture was added to the prepared cell plate. After 6 h, the medium was replaced with fresh complete medium and maintained for 24 h. The cells were then harvested and subjected to subsequent experiments.

### 2.7 Western blot analysis

The cells were lysed on ice using precooled 1 × EBC lysis buffer solution (20 mM Tris-HCL pH 8.0, 125 mM NaCl, 2 mM EDTA, 0.5% NP-40) containing protease inhibitors (Roche, USA). All the samples were prepared with an amount of about 30 micrograms of protein for each lane, and then the proteins were separated via SDS‒PAGE and transferred to 0.2 μm PVDF membranes (Millipore, USA). The membrane was blocked with 5% nonfat milk. The primary antibodies, including ADAM10 (cat#: 25900-1-AP and cat#: 66620-1-Ig, Proteintech, China), N-cadherin (cat#: 13A9, CST, USA), E-cadherin (cat#: 24E10), MMP9 (cat#: 502095, ZENBIO, China), Nocth1 (D1E11, CST, USA), Vimentin (cat#: 289517, Invitrogen, USA), Flag (cat#: F3165, Sigma‒Aldrich, USA), and *β*-actin (cat#: 66009-1-Ig, Proteintech, China) were dissolved in 2% nonfat milk in 1 × TBST, and the membranes were incubated at 4 °C overnight. After the membranes were washed three times with TBST, they were incubated with HRP-conjugated goat anti-mouse (cat#: SA00001-1, Proteintech, China) or goat anti-rabbit (cat#: SA00001-2, Proteintech, China) secondary antibodies at room temperature for 2 hr. Afterwards, the membrane was visualized and quantified via PierceTM ECL Western Blotting Substrate (cat#: 32106, Thermo Fisher, USA). All the experiments were repeated three times.

### 2.8 Proliferation assay

Cell proliferation was monitored via a CCK8 kit (cat#: K1018; APExBIO, USA). H1299 or A549 cells transfected with negative control pcDNA5-FRT-To-2×FLAG-Vector or pCDNA5-FRT-To-ADAM10-2×FLAG-Hyg plasmid, and H1299 or A549 cells transfected with GI254023X (0, 20 μM) were seeded in 96-well plates at 3.5×10^3^ cells/100 μl/well. After 24 h and 48 h of culture, 10 μl of CCK-8 solution was added to each well and incubated at 37 °C for 2~3 h. The intensity of color in the plate was determined at 450 ​nm via a microplate reader (Thermo Scientific Multiskan GO, USA).

### 2.9 Transwell assay

A 24-well Transwell chamber (Corning, USA) precoated with Matrigel was utilized for the invasion assay, while Transwell chambers without Matrigel were used for the migration assay. NSCLC cells (5 × 10^4^) were seeded into the upper Transwell chambers without FBS, while the lower chambers contained 15% FBS DMEM for 48 h. The lower surface was fixed with 4% paraformaldehyde for 30 min before being stained with 0.1% crystal violet for 20~30 min. Subsequently, the migrated/invaded cells on the lower surface of the insert were captured and counted. Each experiment was carried out in triplicate wells and repeated no less than three times.

### 2.10 Wound healing assay

NSCLC cells were seeded in 6-well plates that were pretransfected with negative control pcDNA5-FRT-To-2×FLAG-Vector or pCDNA5-FRT-To-ADAM10-2×FLAG-Hyg plasmids to form a confluent monolayer, after which a 200 μl pipet tip was used to scrape and create an empty gap. After that, the media was replaced with 2% fresh media. The cells were microscopically imaged at 0, 6, 12, 24, 36 and 48 h, and the rate of wound healing was calculated according to the following equation:



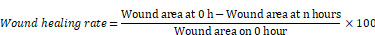



### 2.11 Statistical analysis

All the data are expressed as the means ± S.Ds. and were analyzed via either one-way analysis of variance or two-tailed unpaired Student's *t* tests. For each parameter of all the data presented, “*” *P*<0.05, “**” *P*<0.01.

## 3. Results

### 3.1 ADAM10 overexpression in lung cancer

**Figure 1:**
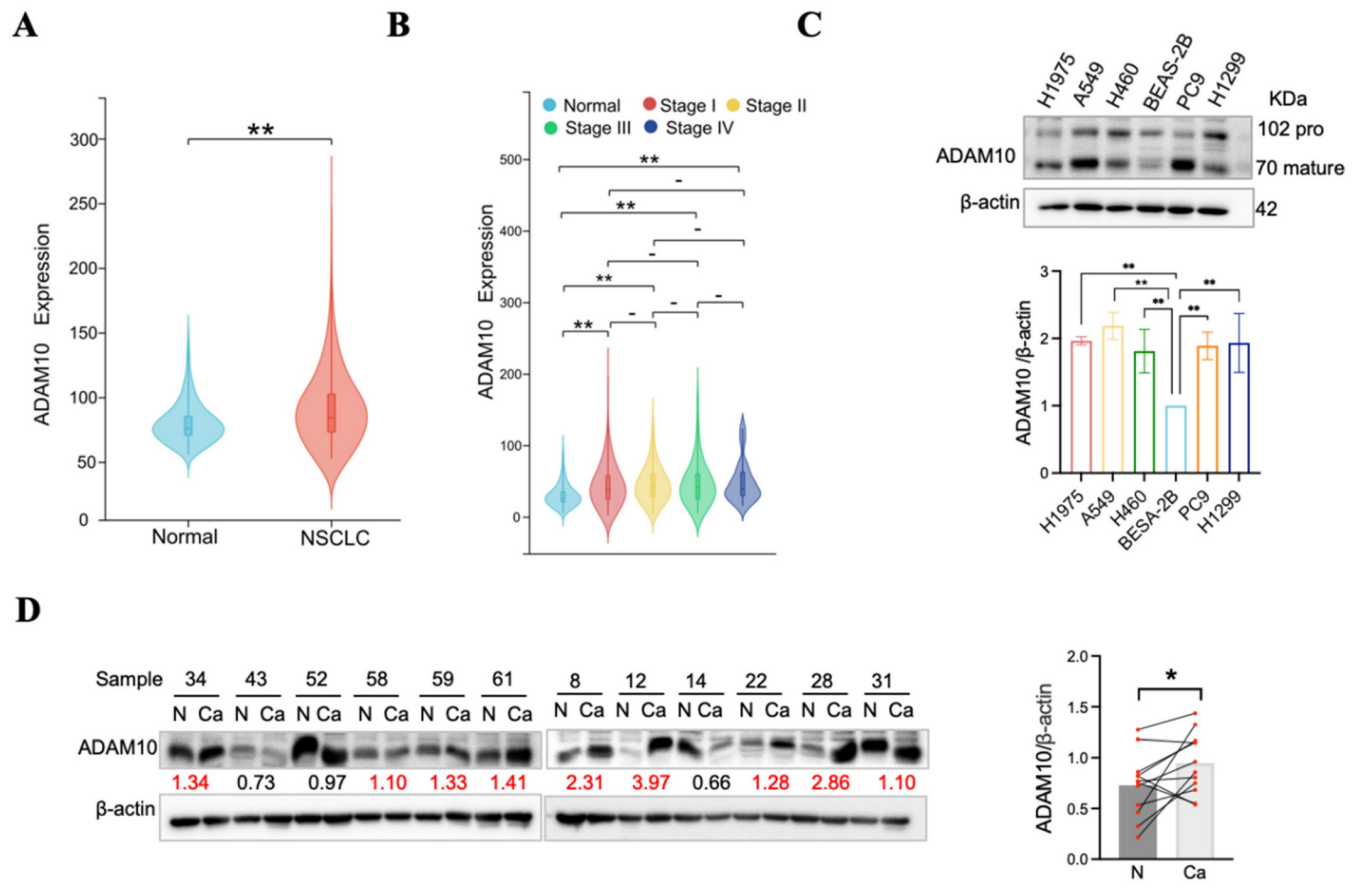
** ADAM10 expression may increase in lung cancer tissues**. A. ADAM10 expression may increase in the non-small cell lung cancer (NSCLC) tissues by TCGA analysis. B. ADAM10 expression may increase in the different stages of NSCLC tissues. C. Western blot of ADAM10 expression increase in five human NSCLC cell lines compared with the normal human bronchial epithelial cell line. Quantitative results for ADAM10 expression. D. Western blot of ADAM10 expression may increase in the Chinese lung cancer tissues and paired adjacent non-cancerous tissue. The number beneath each pair of bands in the blots indicates relative expression ratios. Ratios less than 1 signify decreased expression, a ratio equal to 1 represents unchanged expression, and a ratio greater than 1 indicates increased expression in cancerous samples. (N, adjacent non-cancerous tissues; Ca, cancerous tissues;). And quantitative results for ADAM10 expression in the Chinese lung cancer tissues.

To assess the prevalence of ADAM10 expression in human non-small cell lung cancer (NSCLC), we utilized combined data from the TCGA and GTEx repositories, as well as TCGA tumor tissue data. Initial examination of ADAM10 expression via TCGA datasets revealed significantly elevated levels of ADAM10 in NSCC tissues relative to matched normal tissues (Figure 1A). Furthermore, analysis of ADAM10 expression variation in different clinical NSCC stages versus normal tissues (Figure 1B) revealed the substantial role of ADAM10 in the progression of lung cancer. Specifically, ADAM10 expression was robustly elevated in stage III tumors.

To validate these findings, ADAM10 expression was assessed in five NSCC cell lines and a normal human bronchial epithelium cell line (BEAS-2B). Notably, ADAM10 expression was notably increased in NSCC cell lines (H1975, A549, H460, PC9, and H1299) but not in BEAS-2B cells (Figure 1C). In parallel, twenty-four Chinese NSCLC tissues and their matching nontumorous adjacent samples were obtained via Western blotting. Overall, ADAM10 expression was markedly increased in NSCC, with 9 out of 12 NSCC tissues presenting increased ADAM10 levels, 2 presenting decreased ADAM10 levels, and 1 presenting unchanged ADAM10 levels compared with adjacent normal tissue samples (Figure 1D). Our investigation corroborated the bioinformatically analyzed data.

### 3.2 High ADAM10 expression is positively correlated with poor survival

**Figure 2:**
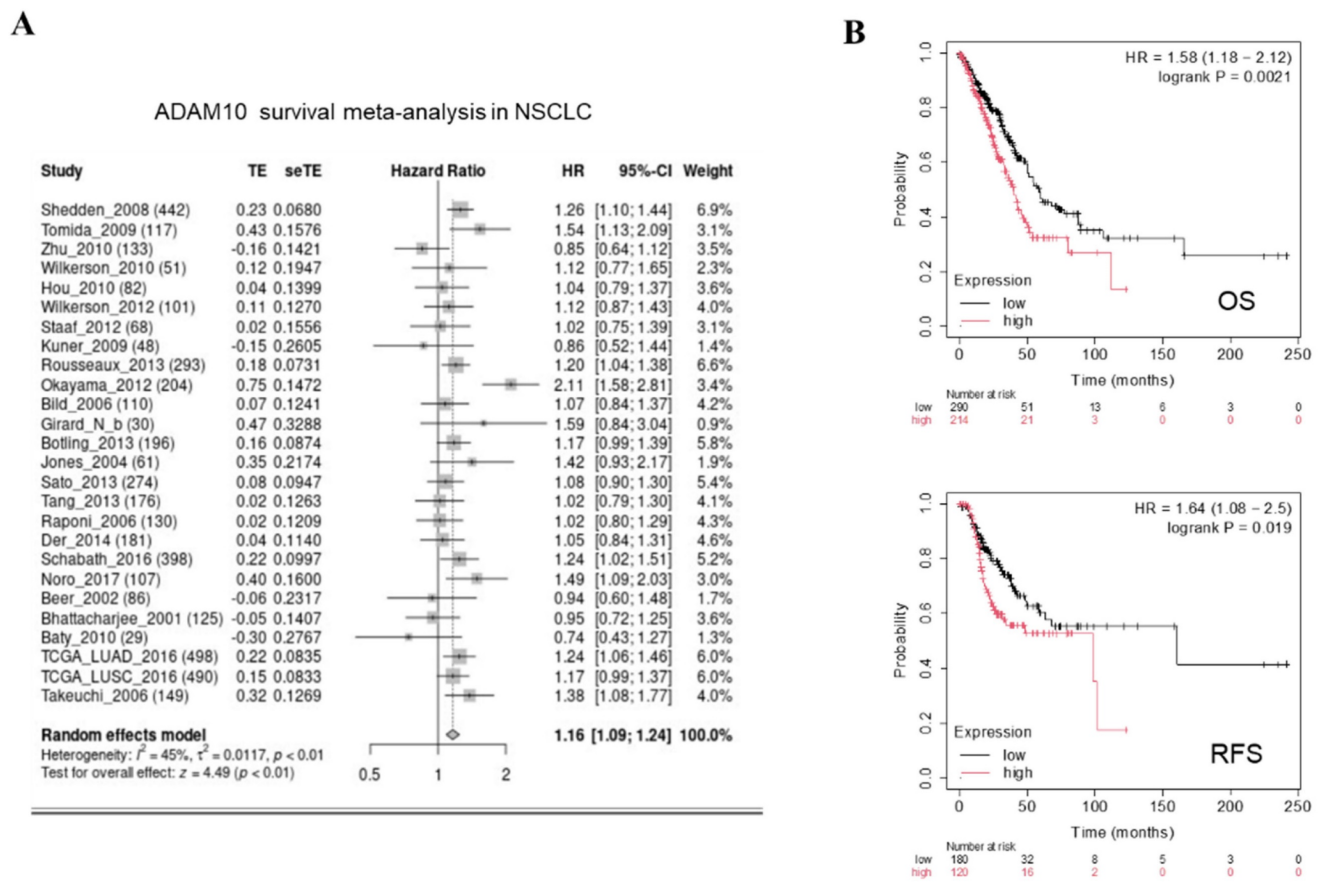
** High ADAM10 expression positively correlated with poor survival**. A. Survival for ADAM10 expression in NSCLC by meta-analysis. High ADAM10 expression has significant association with positive survival outcomes in NSCLC patients. B. Overall survival (OS) and (relapse-free survival) RFS for ADAM10 expression in NSCLC by Kaplan-Meier Plotter. CI indicates confidence interval, SMD indicates standardized mean difference, seTE indicates standard error of treatment effect, HR indicates hazard ratio, TE indicates estimated treatment effect.

To examine whether there is an association between ADAM10 overexpression and reduced survival in NSCLC patients, we performed a meta-analysis separately to explore the relationship between ADAM10 expression and survival in NSCLC patients. The outcome of this analysis revealed that, indeed, patients with high ADAM10 expression had significantly shorter overall survival than those with lower ADAM10 expression in NSCLC, as depicted in Figure 2A. Taking this finding into account along with other relevant literature, it can be deduced that high ADAM10 expression is positively correlated with poor survival in NSCLC patients.

We subsequently focused on assessing the impact of ADAM10 expression on OS and RFS in NSCLC patients. To this end, we applied Kaplan‒Meier plotter analysis, which revealed that patients with high ADAM10 expression experienced significantly shorter OS and RFS than did those with low ADAM10 expression in NSCLC, as outlined in Figure 2B. These findings support our initial conclusion that ADAM10 overexpression is negatively related to OS and RFS in NSCLC patients.

### 3.3 ADAM10 overexpression promotes lung cancer growth, migration, and invasion

**Figure 3:**
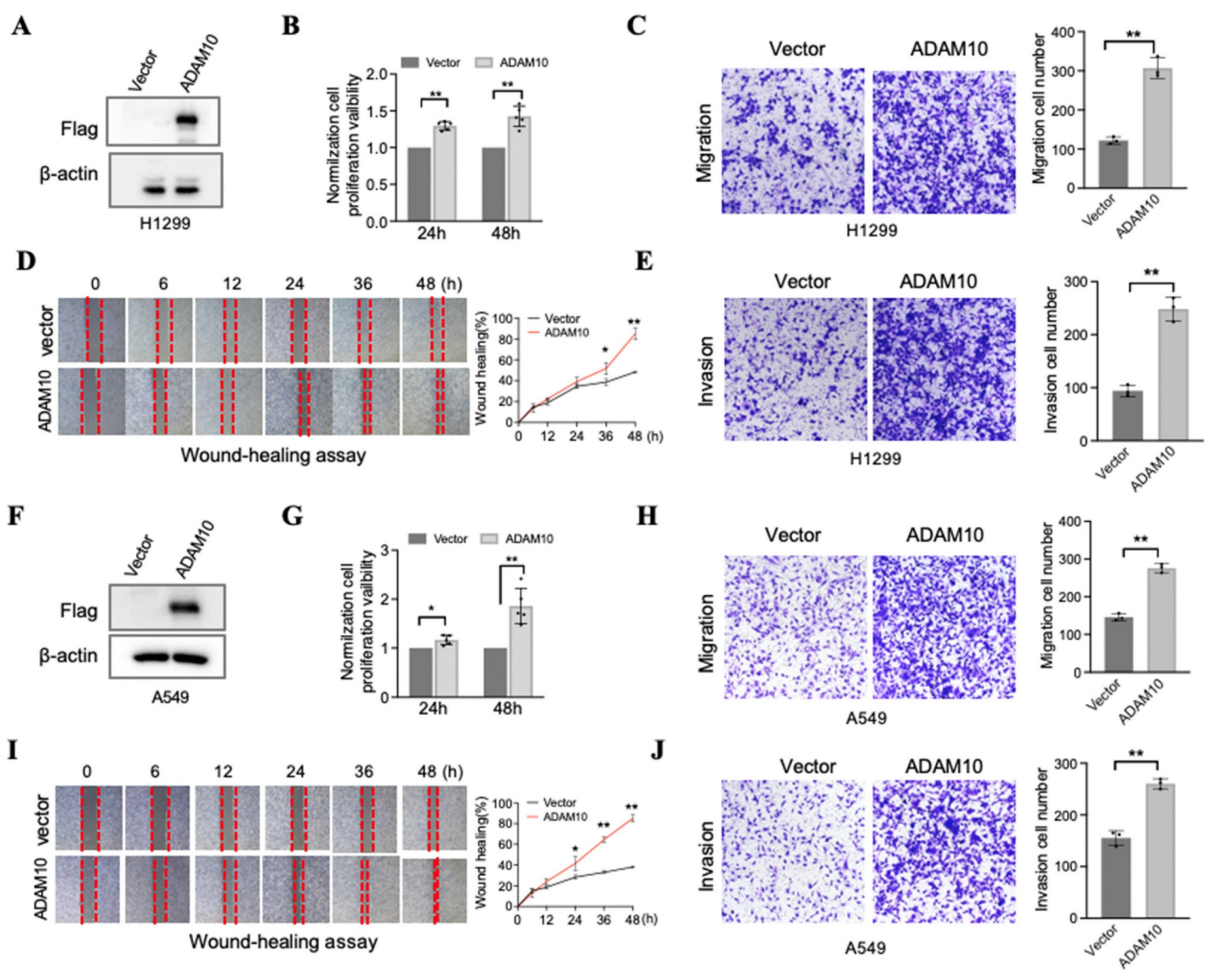
** Overexpression for ADAM10 promotes growth, migration, and invasion in lung cancer cells**. A & F. Overexpression for ADAM10 in lung cancer cells H1299 and A549, respectively. B & G. Overexpression for ADAM10 promotes growth in lung cancer cells H1299 and HA549, respectively. C & H. Overexpression for ADAM10 promotes migration in lung cancer cells H1299 and A549 (magnification: X100), respectively. D & I. Would healing assay shows overexpression for ADAM10 that promotes migration in time-dependents in lung cancer cells H1299 and A549 (magnification: X40), respectively. E & J. Overexpression for ADAM10 promotes invasion in lung cancer cells H1299 and A549, respectively.

Although Guo and colleagues employed the lung cancer cell line A549, which has successful ADAM10 overexpression and the capacity for cell migration and invasion 11, it would be prudent to conduct further experiments utilizing two additional lung cancer cell lines, namely, H1299 and A549. We report here our findings, which, upon analyzing A549 and H1299 cell lines with elevated ADAM10 expression, demonstrate pronounced effects (Figure 3). In the H1299 cell line, when ADAM10 was successfully expressed (Figure 3A), cell growth (Figure 3B), migration (Figure 3C), and invasion (Figure 3D&E) were promoted. This inhibition may be time dependent according to the results of the wound healing assay (Figure 3E). Moreover, results similar to those of H1299 cells were observed in the A549 cell line (Figure 3F-J).

### 3.4 Inhibition of ADAM10 prevents cell migration and invasion in lung cancer

**Figure 4:**
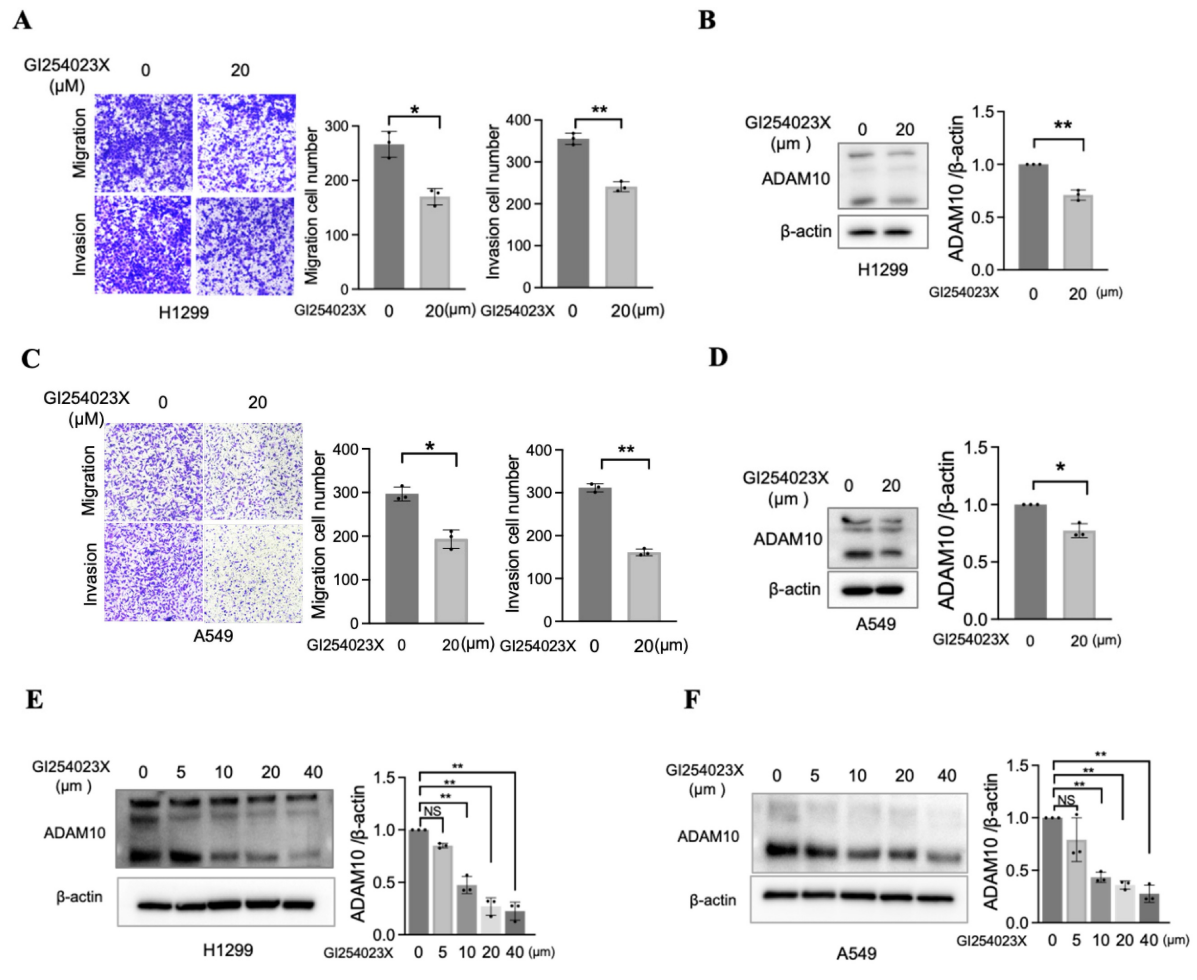
** Inhibition of ADAM10 prevents migration and invasion in lung cancer cells.** A & C. Inhibitor GI254023X for ADAM10 prevents migration and invasion in H1299 and A549 cells (magnification: X100). * P<0.05, ** P <0.01. And quantitative results. B & D Expression level for ADAM10 in H1299 and A549 cells. And quantitative results. E & F. ADAM10 inhibitor (GI254023X) exhibits dose-dependent effect in H1299 and A549 cells. Right lanes: The quantitative results.

Although a previous report established that the overexpression of ADAM10 is correlated with enhanced migratory and invasive capabilities in a lung cancer cell line (A549) 11, further investigations using alternative methods to inhibit ADAM10 are needed. One approach involves the use of small-molecule inhibitors specifically designed to target ADAM10, such as GI254023X 18, 19. To test the efficacy of this inhibitor in blocking ADAM10 function, we treated two lung cancer cell lines, H1299 and A549, with varying amounts of GI254023X. As expected, the migration and invasion of H1299 and A549 cells were significantly inhibited when they were exposed to sufficiently high concentrations of the inhibitor (Figure 4A&C). Concomitant with this decrease in migration and invasion, we observed a corresponding reduction in ADAM10 expression (Figure 4B&D), which was consistent with the inhibitory effect of GI254023X on ADAM10 activity. GI254023X, an inhibitor of ADAM10, effectively reduced the expression level of ADAM10 in a dose-dependent manner in NSCLC cells (Figure 4E&F).

### 3.5 Mechanistic roles of ADAM10 expression in lung cancer development

**Figure 5:**
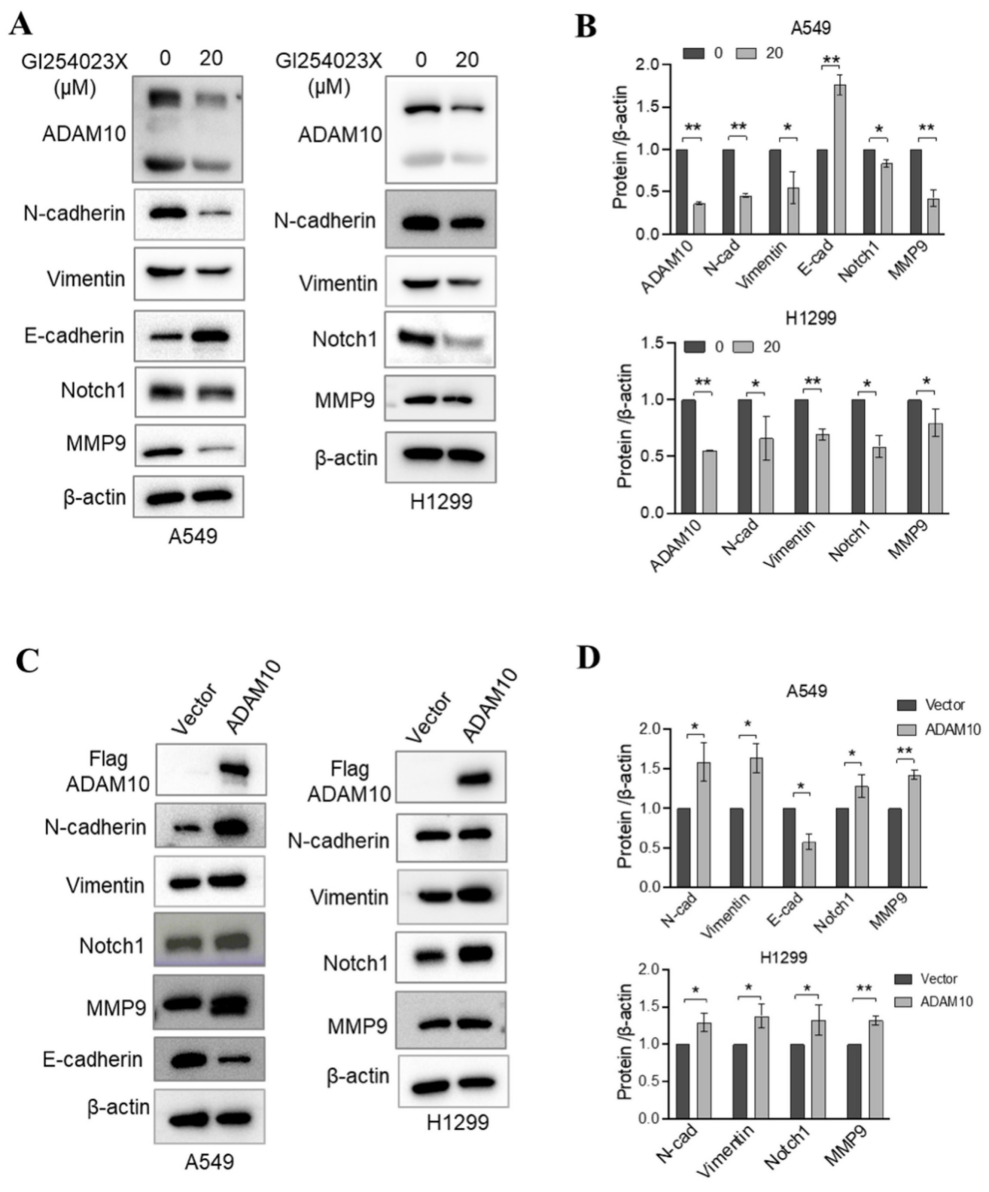
** ADAM10 expression and EMT regulation in lung cancer cell lines**. A. ADAM10 inhibitor GI254023X regulates the expressions of EMT markers and Notch1, MMP9. B. Quantitative results for panel A. C. Overexpression of ADAM10 regulates the expressions of and EMT markers and Notch1, MMP9. D. Quantitative results for panel C. *, *P*<0.05, **, *P*<0.01.

Lung cancer progression and metastasis result from multiple interconnected processes, including epithelial‒mesenchymal transition (EMT)^20^. ADAM10 is a zinc metalloprotease that mainly controls cellular adhesion and migration 21. During EMT, cells gain the ability to migrate and invade, adopting a mesenchymal phenotype, manifested through changes in gene expression patterns, including the upregulation of Vimentin and N-cadherin. A rise in N-cadherin levels can reduce cell adhesion, making it easier for cancer cells to detach from and move away from the underlying tissue, thereby increasing their tendency to colonize distant sites (metastisize)^22^. Therefore, we aimed to examine how active ADAM10 might contribute to these EMT-driven changes in lung cancer development. Using the ADAM10 inhibitor GI254023X, we found that it led to increases in E-cadherin expression in A549 cells while simultaneously reducing the levels of both N-cadherin and Vimentin (Figure 5A&B). Conversely, when ADAM10 was artificially overexpressed in A549 and H299 cells, we observed a concurrent decrease in E-cadherin, along with an increase in both N-cadherin and Vimentin (Figure 5C&D). The expression of E-cadherin is lower in poorly differentiated tumors compared to well-differentiated ones 23-25. Consistent with this, we observed a negative expression of E-cadherin in the H1299 cell line, further corroborating previous findings. These findings reveal novel molecular mechanisms by which ADAM10 expression might play a crucial role in regulating lung cancer progression and could provide targets for new therapeutic strategies against lung cancer.

In addition to participating in lung cancer progression, Notch signaling is involved in EMT induction 26. Notably, ADAM10 plays a critical role in the activation of Notch1 during T-cell development and cancer cell migration and invasion 11, 27. In osteosarcoma, ADAM10 was reported to promote cell growth, migration, and invasion via E-cadherin/β-catenin signaling, including MMP9 28. Thus, we investigated whether changes in ADAM10 expression affect Notch signaling and MMP9 expression through inhibition or overexpression experiments in A549 and H1299 cells. Using western blotting, we found that inhibiting ADAM10 expression via the administration of the inhibitor GI254023X resulted in a decrease in both Notch1 and MMP9 expression (Figure 5A & B). Moreover, we observed increases in the Notch1 and MMP9 expression levels when ADAM10 was overexpressed (Figure 5C & D). These results imply that ADAM10 might act as a positive regulator of Notch signaling in certain contexts and that blockade of its function can lead to a decrease in Notch activity.

## 4. Discussion

Despite progress in understanding the biology of lung cancer, the disease remains a major cause of cancer-related deaths worldwide, with major risk factors including smoking, air pollution, and genetic susceptibilities 29. Several downstream signaling pathways are often activated in various forms of lung cancer, including the PI3K/AKT/mTOR, MAPK/ERK, MEK/ERK, JAK/STAT, NF-κB, and transforming growth factor-beta (TGF-β) signaling pathways 30. Our present study demonstrated the pivotal role of ADAM10 in lung cancer progression, indicating its participation in promoting cell proliferation, migration, and invasion.

More recent studies suggest that ADAM10 contributes to tumorigenesis and progression, suggesting that ADAM10 is a promising new target for cancer therapy 4. ADAM10 is expressed at elevated levels in numerous malignant tumors, including glioblastoma, breast cancer, oral squamous cell carcinoma, Hodgkin lymphoma, non-Hodgkin lymphoma, and multiple myeloma, and its excessive expression is correlated with poor prognosis and distant metastasis in tumor patients 4. Our current analysis of data from the TCGA database and 12 pairs of Chinese lung cancer and adjacent tissue samples revealed that ADAM10 expression was significantly higher than that in matched normal tissues across all stages of lung adenocarcinoma (LUAD) and lung squamous cell carcinoma (LUSC) and during the different lung tumor stages. Despite having only a limited number of Chinese samples (n=12 pairs), examination of the TCGA database revealed that ADAM10 expression reached its highest level in Stage III non-small cell lung cancer (NSCLC) patients. Furthermore, positive correlations were observed between high ADAM10 expression and poor overall survival (OS) and recurrence-free survival (RFS) in patients. ADAM10 is implicated in numerous biological processes, including cell proliferation, invasion, metastasis, and cell adhesion 4-7; these functions have also been observed in prostate cancer 8, uveal melanoma progression 9, and other types of cancers. Further analysis demonstrated that the overexpression of ADAM10 leads to increases in the growth, migration, and invasion of H1299 and A549 lung cancer cells; conversely, the suppression of ADAM10 inhibits these processes in these same cells 11. Our results regarding the role of ADAM10 in lung cancer cell behavior are consistent with those reported by Guo and colleagues 11, who also reported that ADAM10 inhibits cell migration and invasion in the A549 cell line.

The elevated expression of ADAM10 in cancer cells intensifies its function as a sheddase, leading to the release of various cell surface proteins, including ligands and receptors belonging to the Notch, Eph, and erbB families. The involvement of ADAM10 in the cleavage of Notch receptors, a pathway critical for regulating cell differentiation, proliferation, and survival, implies that ADAM10 plays an important role in lung cancer (NSCLC)^31^. Furthermore, activation of Notch signaling mediated by ADAM10 can promote the maintenance of cancer stem cells (CSCs), which are notorious for their resistance to therapy and ability to start metastasis. Hence, this process initiates signaling pathways indispensable for tumor development and perpetuation.

The results of the present study suggest that the ADAM10 protein is involved in modulating the expression of Notch1, Matrix Metalloproteinase 9 (MMP9), and Embryonic Marcus Arcellinus Mononuclear Phagocyte (EMT) markers, such as Vimentin and N-cadherin, through its upregulation while concurrently reducing the expression level of another EMT marker, E-cadherin. These findings offer insights into the potential biological effects of ADAM10 in lung cancer progression. Additionally, similar to the findings of Gao et al. 32, ADAM10 cleaves CX3CL1, which promotes epithelial‒mesenchymal transition (EMT) in liver cancer cells and thereby aids in the migration of these cells, contributing to the process of cancer cell dissemination. As previously reported 27, Notch1 can enhance the migration and infiltration of circulating tumor cells in lung squamous cell carcinoma (LUSC). Furthermore, there is a positive association between MMP9 expression and lung cancer severity 33. Notably, both Vimentin and N-cadherin expression are significantly linked to elevated lung cell mobility and invasiveness 34, 35.

In future research, we will deeply investigate the regulatory mechanism of ADAM10, such as how its expression is increased in non-small cell lung cancer. Moreover, we will conduct screening and experimental studies of small molecule compounds targeting ADAM10.

Overall, this study demonstrated that ADAM10 functions as a diagnostic, prognostic, and predictive biomarker for lung cancer, indicating its potential as a therapeutic target through modification of ADAM10 levels.

## Figures and Tables

**Figure 6:**
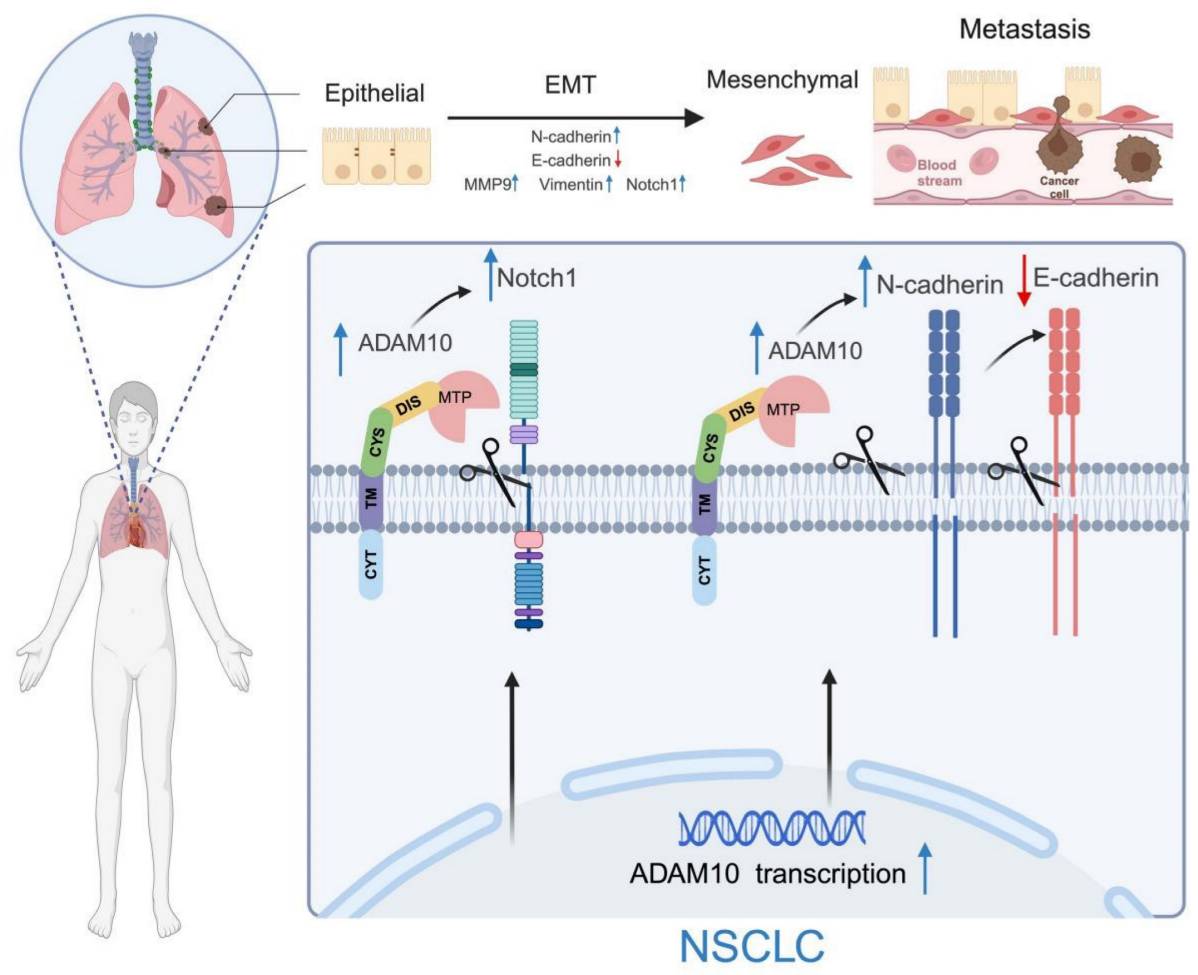
** Working model for ADAM10 in lung cancer.** ADAM10 is overexpressed in lung cancer, resulting in an overall increase in its activity and cleavage of oncogenic substrates such as Notch1, N-cadherin, and E-cadherin, which increases in MMP9 and Vimentin levels. The enhanced activation of the Notch signaling pathway also occurs and ultimately contributes to the EMT development in NSCLC. Note: The figure was drawn with BioRender web-based tool with permission (https://BioRender.com), publication license number: MG27UDUXF4.
